# Asilomar, Gene Cloning’s Origins, and Its Commercial Fate

**DOI:** 10.1007/s10739-025-09803-0

**Published:** 2025-02-18

**Authors:** Doogab Yi

**Affiliations:** Department of Science Studies, Seoul National University, Seoul, Republic of Korea

**Keywords:** Asilomar, Genetic engineering, Risk, Gene cloning, Biotechnology

## Abstract

This paper delves into the historical development of recombinant DNA technology, examining the pivotal controversies surrounding public health and commercialization that emerged with the prospect of gene cloning in the 1970s. The analysis will focus on the recombinant DNA experiments planned, conducted, and aborted by Janet Mertz and John Morrow, two graduate students at Paul Berg’s Laboratory at Stanford University. Their experiments, as I show, served as catalysts for both fear and excitement within the biomedical research community and beyond. This paper begins by reconstructing in some respects Mertz’s and Morrow’s investigative pathways, their contributions to technical developments in gene cloning, and their youthful perspectives on genetic engineering. While Mertz’s initial experimental plan led to the establishment of the Asilomar I Conference in 1973, Morrow’s subsequent cloning experiment, in collaboration with Stanley Cohen and Herbert Boyer, played a crucial role in shifting scientific and public sentiments around recombinant DNA, intensifying the tension between safety concerns and commercial aspirations before, during, and especially after the more famous Asilomar II Conference of 1975. The latter part of this paper briefly examines the commercial fate of early gene cloning within the context of the complex interplay between scientific advancements, societal and public health concerns, and proprietary interests that culminated in Genentech’s cloning of the artificial insulin gene. This paper concludes by discussing how concerns about responsible research practices and biosafety regulation were by the late 1970s increasingly overshadowed by critiques concerning the impact of regulations and academic patenting on scientific competition and laboratory culture.

This paper examines the historical development of recombinant DNA technology and the two related controversies surrounding public health and commercialization that emerged with the prospect of gene cloning in the 1970s. The analysis will focus on the recombinant DNA experiments planned, conducted, and aborted by Janet Mertz and John Morrow, two graduate students at Paul Berg’s Laboratory at Stanford University. Their experiments, as I show, served as catalysts for both fear and excitement within the biomedical research community and beyond. In December 1970, Mertz embarked on a study of the tumor virus, SV40, trying to use recombinant DNA techniques suggested to her by her thesis advisor, Paul Berg. Her proposal to introduce SV40-plasmid recombinant molecules into bacteria, however, elicited biohazard concerns beginning in the summer of 1971, culminating in the first Asilomar Conference on Biohazards on Biological Research (hereafter, Asilomar I) in 1973. While Mertz is often characterized as a neglected graduate student whose brilliant experimental plan was curtailed by the so-called biohazard controversy, she endeavored to approach this issue from a perspective that would balance potential hazards against the advantages of recombinant DNA technology and its use for genetic manipulation of organisms.

This paper posits that Mertz occupied a unique position at the intersection of scientific advancements and societal concerns related to genetic engineering. Mertz was well aware of the potential applications of recombinant DNA technology in the realm of genetic engineering. Her fellow graduate student at Stanford’s Biochemistry Department, Peter Lobban, first envisioned that cells containing recombinant genes could be cloned and express their new genetic information to produce useful therapeutic materials. His 1969 proposal significantly influenced early thinking about the possibilities for genetic engineering (Lobban 1969). Her presentation on the plan to clone SV40 genes in *Escherichia coli* at the summer school at the Cold Spring Harbor Laboratory (CSHL) in 1971, as this paper seeks to demonstrate, reflected not only key technical developments in animal virology but also the growing excitement and apprehension surrounding the prospects of genetic manipulation and its social, cultural, and ethical implications.

In the summer of 1973 Mertz’s fellow graduate student in Berg’s Laboratory, John Morrow, conducted a critical experiment of gene cloning with Stanford geneticist Stanley Cohen and University of California, San Francisco (UCSF) biochemist Herbert Boyer—an experiment Mertz could not perform due to biohazard concerns. While existing scholarship on genetic engineering tends to focus on Cohen and Boyer’s experiments on the replication of bacterial plasmid DNAs across species in bacteria, this paper underlines that it is critical to appreciate Morrow’s cloning and expression of vertebrate DNA in bacteria and the resulting shifts in sentiments around recombinant DNA technology. This pivotal experiment generated strong reactions, intensified by the tension between safety concerns and commercial aspirations within the field of genetic engineering (Echols 2001; Hughes 2001; Yi 2015). At one level, Morrow’s experiment magnified concerns in the scientific community that genetic recombination experiments could create public health hazards by allowing dangerous genes, such as cancer-causing or drug-resistance genes, to cross species boundaries and to escape the lab. This development led participants in the much-publicized Asilomar Conference on Recombinant DNA Molecules of 1975 (hereafter, Asilomar II) to call for a voluntary pause of and guidelines on recombinant DNA research (Berg et al. 1974; Krimsky 1982; Wright 1994; Campos 2024a).

At another level, Morrow’s experiment encouraged entrepreneurial ambitions to use gene cloning technology for medical and commercial development. Morrow’s work demonstrated not just the expression of animal genes through recombinant DNA technology for the first time, but also the cloning of selected DNA segments. His experiment suggested that gene cloning could transform bacteria into biological factories, capable of synthesizing novel pharmaceuticals and other valuable chemicals. The suspension of certain classes of experiments, as this paper demonstrates, complicated the determination of priority and inventorship in gene cloning, as well as the strategies for commercialization. Famously, just prior to Asilomar II in November 1974, Stanford University filed a patent on basic techniques for recombinant DNA work, and Boyer subsequently pursued their commercialization, leading to the founding of Genentech in 1976 (Hughes 2011; Yi 2011). Notably, Berg, one of the co-organizers of Asilomar II, and his graduate student Morrow, were already aware of these efforts to patent the technology. This paper elucidates how Stanford biochemists’ reservations and critics compelled Stanford and Cohen to justify the patenting of recombinant DNA technology amid the moratorium and biosafety concerns.

Many analyses of Asilomar have applauded the unprecedented action of scientists voluntarily halting their research on recombinant DNA technology until the associated risks could be more comprehensively assessed. Some, however, have critiqued the conference participants for adopting an overly technocratic approach to risk assessment, excluding broader ethical, moral, and social considerations (Krimsky 1982; Weiner 2001; Hurlbut 2015; Botelho 2019). The conference thus has been criticized for its exclusionary nature, failing to incorporate diverse social groups and stakeholders who might be affected by the technology. One scientist who participated in Asilomar later criticized the lack of self-reflection that went beyond technical risk, lamenting that “it did little or nothing about the broader issues of the impact of big-business-driven biotechnology on the universities and society in general” (Gisler and Kurath 2011, p. 228).

My reconstruction of Mertz’s and Morrow’s investigative pathways, their contributions to technical developments in gene cloning, and their youthful perspectives on genetic engineering aims to provide a vantage point for understanding the emergence of public health and social concerns, as well as the burgeoning commercial ambitions, surrounding this field in the early and mid-1970s. While Asilomar II ostensibly centered on developing a consensus about dealing with these hazards that would become central to government guidelines for responsible research in the field of recombinant DNA, underlying commercial interests were exerting an increasingly powerful influence on the fate of gene cloning and its applications. Morrow’s subsequent cloning experiment in collaboration with Cohen and Boyer, as this paper shows, played a crucial role in shifting scientific and public sentiments around recombinant DNA, intensifying the tension between safety concerns and commercial aspirations before, during, and especially after Asilomar II of 1975.

Stanford University, in filing its broad recombinant DNA technique patent, claimed that patenting served as a mechanism to control and regulate the use of the technology for biosafety (as had often been claimed before; Creager 1999; Wellerstein 2008). The intense, commercially-driven cloning races that followed Asilomar (featuring, for example, the breach of Asilomar II-born regulations in UCSF’s cloning of insulin) provide little evidence that use of that technology was in fact effectively regulated (Hall 1987). The later part of this paper briefly examines how the outcome of the race to clone insulin, at least for commercial and practical purposes (Hughes 2011; Rasmussen 2014), was partly determined by the complex interplay between scientific advancements, societal and public health concerns, and proprietary interests. This paper concludes by discussing how, by the late 1970s, worries about responsible research practices and biosafety regulation were increasingly overshadowed by critiques concerning the impact of regulations and academic patenting on scientific cooperation and laboratory culture.

## Janet Mertz’s Encounter with Genetic Engineering

In existing accounts of the history of the Asilomar conferences, Mertz is often merely described as a young graduate student who developed the methods needed to perform Berg’s planned experiment to clone a cancer-causing gene (Krimsky 1982; Wright 1994). Her disclosure at the 1971 summer school on Animal Cells and Viruses at the CSHL that she was planning to put genes from a tumor virus, SV40, into *E. coli*, triggered biohazard concerns that ultimately derailed her planned experiment. Her contributions, however, extend beyond this simplified narrative. Mertz’s subsequent experiment on SV40, though never fully executed due to its potential hazards, illustrated the technological advancements that underpinned gene cloning. Mertz’s contributions were not recognized, in that the method patents for recombinant DNA cloning were ultimately secured by more senior scientists, Cohen and Boyer (Yi 2015). Mertz’s subsequent departure after her PhD for England to pursue other research tools for tumor virology, driven by her concerns about the social and ethical implications of genetic engineering, further marginalized her role in the development of recombinant DNA.

Mertz, as this paper intends to show, is a crucial figure for understanding the fuller scientific and sociocultural contexts in which technologies of recombinant DNA research first emerged, and for the discussions prompted by early developments in genetic engineering. Understanding her scientific formation, her choices, and her perspective sheds light on the question of why voices like hers were largely excluded from the contemporary debates on the technical dimensions of risks involving recombinant DNA techniques, including Asilomar II, and how the subsequent commercialization of what became known as genetic engineering eventually proceeded.

To appreciate the significance of Mertz’s role, it is essential to consider the pivotal events of November 1969 that introduced her to genetic engineering. The year 1969 marked two very important events in the history of genetic engineering. One had to do with the public discussion of gene manipulation. In November 1969, Jim Shapiro and Jonathan Beckwith at Harvard University isolated a single gene from the *lac* operon in bacteria, indicating that this achievement could be the first step toward genetic engineering. Instead of promoting their achievement, they warned that the public needed to be informed about the potential detrimental social, cultural, and ethical implications of human genetic manipulations (Shapiro et al. 1969).^1^ The other event had to do with the scientific and technological advances that underlie genetic engineering. In his PhD proposal in November 1969, Peter Lobban, a biochemistry graduate student at Stanford University, put forward the idea of recombining two different genes. More remarkably, he proposed that these recombinant genes could be used not only to create gene clones for use in a variety of basic biomedical research, but also for genetic engineering applications that would allow the mass production of useful gene products, such as human hormones (Lobban 1969).

Mertz can be seen as a figure at the intersection of these two developments. As a student at MIT, she attended a course taught by the biologist Ethan Signer, whose interest in genetic engineering was sparked by public debates surrounding the isolation of the gene by Shapiro and Beckwith. *The New York Times* even featured articles that echoed these concerns, suggesting that manipulating DNA, the fundamental substance of heredity, could lead to frightening consequences.^2^ There was a rising tide of worry, particularly among scientists and activists, about the potential implications of the newfound ability to isolate and manipulate genes. During this time, a group of Boston-area scientists who were aligned with the political climate of the 1960s, including their support for the civil rights movement and opposition to the Vietnam War, formed a group called Science for the People (Moore 2008; Schmalzer et al. 2018). These scientists engaged in a lively debate about what it meant to do science for the people, raising ethical questions about the exploitation of life forms for commercial gain and military purposes (Beckwith 2002; Botelho 2019). Biologists, including Beckwith himself, expressed concern regarding the potential for eugenic applications of genetic engineering to exacerbate existing social and racial disparities.

Mertz attended Signer’s class at MIT in which he discussed rapid developments in technologies of gene manipulations such as the ability to move virus DNAs into bacterial cells, and how these gene manipulation technologies could lead to therapeutic, eugenic, and military applications.^3^ Signer developed his discussions on genetic engineering into a paper, in which he cited a 1968 article on the insertion of mammalian viruses like SV40 into the chromosomes of bacterial cells, and discussed its use for gene therapy, manufacturing drugs, and the cloning of desired embryos (Sambrook et al. 1968). Signer in turn cautioned that marginalized and economically disadvantaged groups had historically been disproportionately subjected to human experimentation and organ donation, noting a prominent recent publication on the ethics of human subjects research (the spring 1969 special issue of *Daedalus* on that theme of). He expressed concern that advancements in genetic manipulation could exacerbate the vulnerability of these marginalized populations to exploitation. He called for a social discussion on “who decides how and to whom gene manipulation is to be applied” (Signer 1974, p. 221). Mertz, who the sociologist Sheldon Krimsky characterized as a “MIT-type middle-of-the-road radical” (Krimsky 1982, p. 28), was deeply influenced by the discussions around genetic engineering and became increasingly aware of the implications of her research in molecular biology.

Mertz, a 1970 MIT graduate, became only the third graduate student who was a woman to join the Biochemistry Department at Stanford University. In December of that year, she joined Berg’s Laboratory, where his group was pioneering the artificial synthesis of recombinant DNA to investigate the mechanisms underlying tumorigenesis by the SV40 virus. The research in Berg’s Lab closely aligned with Lobban’s concurrent research to construct a transducing virus, i.e., a recombinant DNA capable of transferring and expressing genes across host species. Lobban, a graduate student in Dale Kaiser’s Laboratory in the same department, tried to capitalize on an observation that bacterial viruses (i.e., phage) frequently transfer genes from other bacteria to *E. coli* hosts, offering a valuable tool for studying gene expression. He hypothesized that by recombining phage DNA with a gene of interest, he could insert the gene into a host cell via phage infection and subsequently express it. As Lobban stated in his proposal, an “eventual goal for the method [of joining two DNA molecules …] would be to produce a collection of transductants synthesizing the products of genes of higher organisms” (Lobban 1969, p. 13). He further articulated that “if the bacterial host of the transducing genomes [bearing mammalian genes] is able to transcribe and translate them, it could be used as a source of the gene product that might be far more convenient than the mammalian cells themselves” (Lobban 1972, pp. 128–129). Lobban clearly envisioned recombinant DNA technology as a means to transform host cells into biological factories for manufacturing medically valuable gene products, such as antibodies and human hormones (Yi 2015).

Mertz’s prior exposure to discussions concerning the scientific and societal implications of genetic engineering equipped her with a relatively comprehensive awareness of the potential ethical risks and benefits associated with this field as she commenced her doctoral research on SV40. Alongside her in Berg’s Lab was David Jackson, a postdoctoral fellow who in autumn 1969 embarked on the artificial construction of a gene-transducing system by recombining SV40 DNA with a bacteria plasmid, λdv*gal*, with the goal of exploring whether bacterial genes might function in mammalian cells. Mertz’s research project aimed to replicate mutant portions of the SV40 genome in an *E. coli* host using the recombinant DNAs created by Jackson, with the goal of studying the biological functions of SV40 genes at a molecular level when cloned and then reintroduced back into mammalian cells. Mertz wanted to understand how expression of genes is regulated in mammalian cells and to explore the roles of SV40 genes in tumorigenesis. First, she needed to test whether SV40 DNA could be cloned and, possibly, expressed in *E. coli*. A successful outcome would not only contribute to cancer research but also advance the field of genetic engineering as envisioned by Lobban. Reflecting on her decision in 1977, Mertz acknowledged that she had reservations about the potential implications of her research. However, she ultimately chose to pursue the SV40 project, motivated in part by its potential medical benefits for cancer research and in part by her belief that “being able to put some DNA into *E. coli* is still a long way from being able to change people’s genes.”^4^

## Biohazards and Mertz’s Aborted Cloning Experiment

In December 1970, Mertz began to work on experiments to insert the SV40 gene into a plasmid vector, starting to construct a plasmid that would replicate inside a new bacterial host. She started by collaborating with Douglas Berg, a postdoctoral fellow in Kaiser’s Lab, to isolate λdvgal 120, a plasmid that contains the entire *gal* operon of *E. coli*. The presence of the *gal* DNA would enable scientists to identify bacteria that contain this cloning vector when it is transferred back into its bacterial host. Recognizing her need for lab expertise in culturing and manipulating SV40 in animal cells, Berg wrote a letter of recommendation to Robert Pollack in March 1971, so that Mertz could attend his summer course at CSHL. As Berg wrote, “I’d like to strongly support her request inasmuch as her present research work and that which she will be engaging in over the next three or four years will deal with animal cells in culture and the transformation by polyoma and SV40... She is very sophisticated in molecular biology but has little experience in the cell culture methodology. The course at Cold Spring Harbor would be extremely helpful to her in getting on with her work.”^5^ By the time Mertz headed to the CSHL in June 1971, she had successfully isolated λdvgal 120 and shown that its purified plasmid DNA could be reintroduced back into *E. coli* using a calcium method that had recently been developed in Kaiser’s Lab, thereby laying the groundwork for creating λdvgal 120-SV40. It meant she had a technique for putting manipulated DNA into an *E. coli* host and reestablishing it as a plasmid.

During the three-week course on animal viruses and cell culture techniques, Pollack facilitated a general discussion concerning the safety considerations associated with working with these materials. Mertz mentioned her research to the class, outlining her intention to recombine SV40 genes with her bacterial plasmid and thus clone the SV40 genes in *E. coli*. Demonstrating an understanding of the potential applications of recombinant DNA technology, Mertz highlighted the feasibility of this approach, asserting that her laboratory at Stanford was “now almost at the point where we should be able to take mammalian DNA’s [sic], replicate them in *E. coli*, and then have these DNAs [sic] as clones for use in a variety of experiments.” As she informed Pollack and her classmates at the CSHL, “we were almost on the verge of genetic engineering.”^6^

While Mertz approached this issue from the perspective of weighing the potential hazards against the benefits of genetic engineering, Pollack immediately expressed grave concerns about the prospect of new strains of *E. coli* rendered more pathogenic by expressing SV40 genes. Pollack underscored the potential public health risks associated with Mertz’s proposal to introduce SV40 genes into *E. coli*, a bacterium residing in the human gut. He vehemently insisted that the experiment be terminated immediately, contacting Berg to emphasize the urgent need to discontinue research that could inadvertently result in the cloning of a cancer-causing gene and its dissemination within the human population (Krimsky 1982, p. 31). Mertz regarded Pollack’s concerns as a hypothetical risk she had not seriously considered previously, and became uneasy with working on genetic engineering, with its complex and vexing ethical, moral, and social implications. She recollected that she was initially thinking about “the Shapiro-Beckwith experiment and the controversy that resulted and so forth. I pretty much held the radical point of view that we shouldn’t go around developing genetic engineering.”^7^ She told Berg that she would not pursue her original project.

Initially Berg did not see any real danger in the genetic exploration of SV40, and felt his lab’s attempt to recombine and express SV40 genes in bacteria would illuminate tumorigenesis, potentially bringing medically useful research results in dealing with cancer. Meanwhile, in August 1971 Lobban succeeded in joining together two DNA genomes of the bacteriophage P22 in vitro. With the help of Lobban, Jackson then joined SV40 with λdvgal 120 in vitro in October 1971, creating the very first recombinant DNA molecules coming from two totally unrelated species (Jackson et al. 1972). Both researchers leveraged the diverse enzymatic repertoire and technical resources available within the Stanford Biochemistry Department to recombine DNA. In the winter of 1971, however, Berg, in consultation with Stanford geneticist Joshua Lederberg who had been involved in safety discussions on the use of biological and chemical weapons, decided to suspend this line of work in the laboratory. No one in his lab attempted to introduce or replicate this recombinant DNA in either *E. coli* or mammalian cells. After Jackson left for a job at the University of Michigan in early 1972, Berg reassured Pollack that his lab would not try to clone SV40 into *E. coli* (Krimsky 1982, p. 37).

Mertz, having decided not to work on a project that might lead to the development of genetic engineering, shifted her dissertation project to study SV40 by other means. Morrow, her fellow graduate student in Berg’s Lab, showed that *Eco*RI enzyme cut SV40 DNA once at a unique site, making it a useful reference point for mapping SV40 genes (Morrow and Berg 1972). During her experiment in April 1972, Mertz found out that *Eco*RI-cut linear SV40 DNA could also be made back into circular DNA molecules in vitro with very high efficiency by incubation with *E. coli* DNA ligase. Within days of this discovery, in collaboration with Ronald Davis, a beginning assistant professor in the department at the time, Mertz showed that cleavage of any DNA with *Eco*RI generated sticky ends that could bond together in vitro at low temperatures even in the absence of DNA ligase. Mertz immediately realized that creating complementary tails on the ends of DNA molecules by simply incubating these DNAs with *Eco*RI enzyme made it very easy to create recombinant DNAs by mixing together *Eco*RI-cut DNAs and joining them together with DNA ligase. Mertz demonstrated the feasibility of this method by using it to construct SV40-λdvgal 120 recombinants at very high efficiency in early June 1972. In their 1972 paper, Mertz and Davis underscored this breakthrough, claiming “any two DNAs with RI endonuclease [*Eco*RI] cleavage sites can be ‘recombined’ at their restriction sites by the sequential action of RI endonuclease and DNA ligase” (Mertz and Davis 1972, p. 3374). This breakthrough marked a substantial advance in genetic engineering, drastically simplifying the method for synthesis of recombinant DNA molecules.

At the subsequent Tumor Virus Meeting held at CSHL from August 16 to 19, 1972, Mertz reported this result. As she told the audience, the SV40-λdvgal 120 recombinants were created as plasmids that could, in theory, replicate inside either *E. coli* or mammalian host cells. During the Q&A immediately after her talk, someone asked if she had put this recombinant DNA into *E. coli* to see whether she could actually clone SV40 DNA. Mertz answered that she had autoclaved them instead due to biohazard concerns (Yi 2015, pp. 94–97).

## Asilomar I: Oncogenic Viruses and Technologies of Gene Manipulation

Amidst growing concerns among the scientific community regarding the rapid advancements in recombinant DNA research, Berg, in collaboration with other prominent scientists and government officials, convened a meeting in January 1973 at the Asilomar Conference Center in California. Asilomar I was specifically focused on discussing emerging public health risks and laboratory safety procedures associated with recombinant DNA research involving tumor-inducing viruses (Hellman et al. 1973; Krimsky 1982). Asilomar I in a sense was a direct outcome of the rapid advancements in animal virus research, which had gained significant funding and scientific prominence since the late 1960s. The passage of the National Cancer Act in December 1971 further accelerated research into the viral etiology of cancer, emphasizing the importance of animal viruses as model systems (Morange 1997; Gaudillière 1998; Scheffler 2019). Beyond their role in cancer research, animal viruses offered a valuable tool for investigating the molecular genetics of eukaryotic biology. Inspired by the ability of these viruses to transduce genes in mammalian cells, Berg’s foray into cancer research focused on the use of SV40. His 1970 American Cancer Society grant application proposing a method to construct recombinant DNAs in vitro marked a pivotal moment in the field: initially employed as a model for studying tumorigenesis, animal viruses subsequently evolved into an artificial transduction system that could be used to clone genes for exploring eukaryotic biology. The development of this groundbreaking recombinant DNA technology was an outcome of Berg’s strategic adoption of animal virus experimental systems (Yi 2008).

In the early 1970s, concerns arose regarding the potential dangers associated with research involving animal viruses and cells due to the identification of C-type particles (a group of RNA viruses) as potential cancer-causing entities. In 1969, scientists Robert Huebner and George Todaro at the National Cancer Institute (NCI) proposed a provocative hypothesis that all vertebrates might harbor type-C virus genomes, inherited through their shared evolutionary history. Huebner suggested that these ubiquitous endogenous retroviruses could play a role in tumorigenesis. Drawing upon the operon model of gene regulation, they postulated that these viral genes, termed “oncogenes,” might be regulated by host genes and repressors. Huebner and Todaro argued that mutations, carcinogens, genetic defects, or aging could activate these endogenous oncogenes, leading to cancer. They proposed that the activation of oncogenes might not result in virus production but could instead produce specific cancer-causing proteins, ultimately causing cancer. This virogene–oncogene theory of cancer presented a novel perspective, viewing the disease as a biological event triggered by the activation of universal, specific viral “oncogenes” (Huebner and Todaro 1969; Scheffler 2019). Most scientists, as Mertz indicated, discontinued mouth pipetting and began to build safer laboratories for virus research.^8^

Berg framed the biohazard risks associated with recombinant DNA research within the broader context of the oncogene paradigm, which posited animal viruses as potential carriers of cancer-causing genes. In 1971 Berg paid a visit to the NIH campus in Bethesda, discussed his concerns about recombinant DNA experiments with virologists who were working on SV40, visiting the “Memorial Laboratories” of Building 7 on the NIH campus, dedicated to scientists who had contracted fatal disease during research (Fredrickson 2001, p. 9). To address biosafety issues around work with tumor viruses at Asilomar I, Berg invited prominent animal virologists like Andrew Lewis and Wallace Rowe from the NIH, as well as medical researchers from Harvard and Yale Medical Schools. Notable attendees included Al Hellman, James Watson, David Baltimore, Norton Zinder, and Michael Oxman. During the conference, Berg called for “caution and some serious effort to define the limits of whatever potential hazards exist.” He further emphasized the importance of responsible action, stating, “to do less, it seems to me, is to play Russian roulette, not only with our own health, but also with the welfare of those who are less sophisticated in these matters and who depend on our judgment for their own safety” (Hellman et al. 1973, p. 354).

While Asilomar I is often remembered primarily for its focus on biosafety issues, it also explored the potential benefits of animal virus research and the social and ethical implications of using viruses as a technology for genetic engineering. Berg invited Richard Roblin, who had written an article in *Science* about a possibility of gene therapy by manipulating mammalian cells by virus infections (Friedmann and Richard 1972). Berg wanted to discuss some of the benefits that might come out from building an artificial system for manipulating genes through viral transduction. At the same time, Berg began to pay attention to some of the social consequences and moral issues involved in recent advances in genetics and molecular biology that might be used for reproductive cloning and genetic engineering technologies, corresponding with the bioethicist Leon Kass (Krimsky 1982; Crowe 2021, pp. 195–231). Some scientists even warned about the price of precautions and Berg’s voluntary moratorium on recombinant DNA research. During Asilomar I, Francis Black of the Yale University School of Medicine proclaimed that “we are finally discussing the costs of the safety precautions and have come to the realization that they will inevitably reduce the number of grants available and increase the time required to reach our ultimate goal. If we do believe in our mission of trying to control cancer, it behooves us to accept some risk. Even if, as has been suggested, five or ten people were to lose their lives, this might be a small price for the number of lives that would be saved” (Hellman et al., 1973, p. 350; Krimsky 1982, p. 67).

By the conclusion of Asilomar I, Berg initiated a prospective study, collecting blood samples from his lab members. The study aimed to monitor individuals for the development of cancer and to investigate their prior exposures. Berg directed his laboratory staff to undergo testing for antibodies against SV40. The results revealed that all laboratory personnel had “seroconverted,” indicating exposure to the virus (Jones 2013, p. 58). While no cases of illness were reported, Mertz did not want to pursue her recombinant DNA cloning experiment further, even with her discovery that one could easily construct recombinant DNAs using EcoRI and the very real possibility that her λdvgal-SV40 recombinants could enable her to be the very first person to experimentally demonstrate gene cloning. As Mertz recollected, she “was not going to have anything further to do with this project, or, for that matter, with anything concerned with recombinant DNA.”^9^ She felt that “it’s for society to decide whether any given discovery or potential discovery from research is for their benefit or not, not for us, as scientists, to decide.”^10^ Still when the medical and agricultural implications of recombinant DNA technology later gained wider attention, Mertz’s 1972 experiment was considered as a critical prior art of gene cloning that complicated the patenting of early gene cloning (Yi 2015).

## Of Clones and Bacterial Factories

By mid-1972, Stanford biochemists had assembled a critical set of techniques and materials necessary for generating recombinant DNA constructs, introducing these constructs into host cells, and utilizing a viable plasmid to express and propagate them. The discovery of *Eco*RI’s unique DNA cleavage site in SV40 by Morrow facilitated the chemical manipulation of the virus chromosome. Mertz and Davis identified *Eco*RI’s ability to create identical cohesive DNA ends. Boyer’s Lab generously provided the *Eco*RI enzyme that was a key material needed for these studies, with his lab determining the sequence of the *Eco*RI-generated ends. Articles describing all three of these findings were published in the same November 1972 issue of the *Proceedings of the National Academy of Sciences* (hereafter, *PNAS*). During this time, the emerging network of recombinant DNA researchers became aware of Mertz’s experiments and her aborted cloning. The development of recombinant DNA technology for genetic engineering arose from this early network within Stanford’s Biochemistry Department. The suspension of recombinant DNA experiments involving tumor viruses inadvertently provided an opportunity for other scientists, such as Boyer and Cohen, to explore recombinant DNA techniques using nonviral materials for gene cloning (Yi 2015).

Cohen (in the Genetics Department at Stanford located just down the hallway from the Berg Lab) and Boyer, eventually recognized as the legal inventors of recombinant DNA technology, skillfully harnessed the advantages of their plasmid experimental system for gene cloning. This achievement eclipsed the central position that Stanford biochemists had held in early recombinant DNA research. Cohen had been investigating antibiotic resistance plasmids, focusing on how drug-resistance genes integrate into plasmids and confer antibiotic resistance properties to other bacteria. He envisioned utilizing the recombinant DNA technology of Mertz and Davis to clone drug-resistance genes using his fortuitously isolated pSC101 plasmid as a vector to transport these genetic elements.

Cohen and Boyer have attributed the conception of the plasmid experimental system as a novel genetic engineering technology to a scientific conference held in Hawaii in November 1972. During this conference, they discussed the recently published Mertz and Davis technique for constructing recombinant DNA using *Eco*RI. Stanley Falkow, a microbiologist who moved to Stanford in 1981, wrote a supporting letter to confirm that he had been “present when Stanley Cohen and Herbert Boyer discussed their idea for a collaboration.” According to Falkow, Cohen and Boyer’s idea “was to introduce foreign DNA into a plasmid having an antibiotic marker and introduce the resulting hybrid DNA into a bacterial host to see if the hybrid plasmid would be biologically functional”^11^ (Falkow 2001; Campos 2024b). It was Cohen who proposed the collaboration with Boyer, whose lab had become a major source of *Eco*RI and enzymological expertise. Still, the technology was conceived as a research technology for studying antibiotic resistance plasmids at this stage.

In March 1973, just a few months after beginning their collaborative efforts, Cohen and Boyer successfully demonstrated the experimental creation of recombinant plasmids by joining genes from related prokaryotic species in vitro (Cohen et al. 1973). These recombinant plasmids were subsequently propagated in *E. coli* as the host bacterium. Cohen and Boyer’s experiment employed the antibiotic resistance plasmid pSC101, raising concerns about biohazards related to spreading antibiotic resistance traits. Cohen wanted to withhold the news of the experimental success until the publication of their first intraspecies recombinant DNA cloning experiment. Boyer, however, first publicly announced the news of this successful experiment at the Nucleic Acids Gordon Conference held between June 11 and 15, 1973.

Although the possibility of propagating recombinant DNA within bacterial cells had been anticipated and even feared by some scientists, Cohen and Boyer’s 1973 experiment provided concrete evidence of the replication of recombinant DNAs assembled in vitro. Boyer’s presentation at the Gordon Conference detailed the collaborative efforts between himself and Cohen, highlighting the use of the *Eco*RI restriction enzyme to join two distinct drug-resistant plasmids, pSC101 and R6-5. The experimental simplicity of creating recombinant DNA with EcoRI and utilizing plasmids for transporting recombinant DNA into living bacteria raised concerns among some scientists, regarding both genes from animal viruses and drug-resistance genes. These concerns were brought to the attention of Maxine Singer, the co-chairperson of the Gordon Conference, who had a keen interest in the ethical and social implications of molecular biology (Watson and Tooze 1981; Krimsky 1982). With younger scientists raising broad social questions about recombinant DNA technology, Singer and her co-chair, Dieter Söll, wrote a letter to the National Academy of Sciences and the Institute of Medicine, emphasizing the potential risks associated with this technology. They highlighted that “new kinds of hybrid plasmids or viruses, with biological activity of unpredictable nature, may eventually be created [… and] certain hybrid molecules may prove hazardous to laboratory workers and to the public” (Singer and Söll 1973, p. 1114; Krimsky 1982, p. 75). They subsequently requested the establishment of an expert panel to address the biohazard issues related to recombinant DNA technology.

After the Gordon Conference, Morrow, who maintained frequent communication with Boyer regarding his SV40 mapping project using *Eco*RI, considered initiating a collaboration with Boyer. It is worth noting that while Mertz’s experiment had the potential to create new cancer viruses with genes acquired from *E. coli* (i.e. super-SV40), as well as *E. coli* expressing genes from SV40 (i.e. super-bacteria), the Cohen–Boyer experiments presented only the latter (super-bacteria) class of risk. Recognizing the potential of Cohen and Boyer’s replication of DNA fragments from the same bacterial species, Morrow saw an opportunity to pursue the cloning of foreign genes. Morrow was acutely aware of the public health concerns associated with recombinant DNA research, having witnessed the challenges faced by his fellow graduate student Mertz, who had abandoned her SV40 recombinant DNA cloning experiment. Morrow believed that *Xenopus* ribosomal DNA, which he already had in his possession in highly pure form, would be a suitable eukaryotic gene for molecular cloning, as it was much less likely to raise biosafety concerns (Rasmussen 2014, p. 37). With Morrow’s *Xenopus* ribosomal DNA, Cohen’s pSC101 plasmid, and Boyer’s *Eco*RI enzyme, they already had on hand all the necessary reagents to clone this eukaryotic gene. Given that Cohen and Boyer’s initial recombinant DNA experiment was confined to the replication of DNA fragments from related bacterial species, the *Xenopus* cloning experiment, if successful, would represent the first demonstration of the cloning foreign and animal cell genes in bacteria (Yi 2015).

By October 1973, Morrow, in collaboration with Boyer and Cohen, demonstrated that recombinant DNA comprising the pSC101 plasmid and frog ribosomal DNA could be introduced into bacteria and replicate there. Furthermore, they showed that the *Xenopus* DNA was transcribed into RNA, suggesting the feasibility of expressing cloned eukaryotic genes. Berg did not know about Morrow’s experiment with Boyer and Cohen prior to its completion, which would eventually strip his group’s priority in gene cloning and made Berg quite upset. Berg still appreciated the significance of Morrow’s experiment: “[T]he first real notion of cloning comes out of the experiment that John Morrow did together with Herb Boyer and Cohen where they cloned the ribosomal DNA sequences from the frog.” According to Berg, “the introduction of a foreign, totally unrelated DNA, frog DNA, into a bacterial plasmid, introducing it into bacteria and getting the bacteria to propagate these was the first demonstration that you could propagate foreign DNA in bacteria.” More importantly, Berg underlined, “that each colony produced from this was a clone, a clone of the original DNA.”^12^ Morrow presented his *Xenopus* cloning experiment at the Biochemistry Department’s annual meeting in October 1973 (in Asilomar), and many Stanford biochemists were excited about its scientific implications. In November of 1973, Mertz wrote to the MIT scientist Mary Lou Pardue: “The J. Morrow, S. Cohen, and H. Boyer experiments of replicating *Xenopus* r[ibosomal] DNA in *E. coli* by attaching it to an R factor plasmid using RI endonuclease and ligase really work. They even detect transcription of the *Xenopus* rDNA. Think of the possibilities.”^13^

Morrow’s experiment marked a key turning point in the evolution of genetic engineering. The successful transcription of the *Xenopus* DNA into RNA and the fact that each colony (clone) represented a distinct segment from the original DNA mixture demonstrated the feasibility of cloning a foreign gene and producing its corresponding product through genetic engineering (Morrow et al 1974). Berg compared this achievement to Annie Chang’s (Cohen’s technician) concurrent interspecies gene-cloning experiments (Chang and Cohen 1974), downplaying the latter’s accomplishment as merely introducing the drug resistance property of *Staphylococcus aureus* plasmid into *E. coli*, falling short of true interspecies gene cloning.^14^ This meant that Morrow’s eukaryotic gene cloning significantly broadened the applicability of genetic engineering. Morrow’s collaborative work suggested that gene cloning could transform bacteria into biological factories, capable of synthesizing valuable substances and novel pharmaceuticals. This research underscored that gene cloning was not only a pivotal technique for biomedical research and genetic analysis but also held substantial commercial potentials.

## Asilomar II and Commerce

In the meantime, Berg came to be in charge of the organizing committee for Asilomar II after the Singer-Söll letter. On April 17, 1974, the National Academy of Science convened a study panel led by Berg to consider “whether or not there is a *serious* problem growing out of present and projected experiments involving the construction of hybrid DNA molecules in vitro” (Krimsky 1982, p. 82). One of the topics discussed there was Morrow’s groundbreaking animal gene cloning experiment, which demonstrated the potential for expressing any DNA sequence within a plasmid. This significant achievement underscored the pressing need to address the potential risks associated with cloning hazardous genes, such as those implicated in toxicity, infectious diseases, and oncogenesis. Indeed, Morrow’s experiment served as a catalyst for the establishment of a voluntary moratorium, culminating in the drafting of the important Berg letter.

David Baltimore, who learned of Morrow’s gene cloning experiment as a member of Berg’s Asilomar planning committee, communicated this result to *New York Times* journalist Victor McElheny.^15^ McElheny’s article appeared a few weeks before the so-called Berg letter that called for a voluntary deferral of certain types of recombinant DNA research. It did not specifically mention biohazards given that the Morrow experiment was performed with foreign ribosomal DNA, a gene that all organisms possess. Prepared in communication with Stanford’s Joshua Lederberg, Cohen, and Morrow, the *New York Times* article on recombinant DNA technology instead delved into the promising potential of genetic engineering that would make “bacterial factories” to make products from “medically significant animal genes.” The cloning work with animal genes, of which Morrow was the first author, drew more interest from the public and the scientific community. The article noted that “by isolating animal or even human genes […scientists could make] colonies of *Escherichia coli*, equipped with the gene‐carrying plasmids, growing large supplies of insulin for diabetics” (McElheny 1974). Lederberg even claimed that the cloning of animal genes through recombinant DNA technology “may completely change the pharmaceutical industry’s approach to making biological elements such as insulin and antibiotics” (Lederberg 1974). McElheny’s article served as a valuable source for both the scientific community and the public about the potential benefits of recombinant DNA research on both agriculture and medicine.

Niels Reimers, an enterprising technology transfer manager at Stanford’s Office of Technology Licensing (OTL), came upon McElheny’s article, realizing some of the transformative potentials of recombinant DNA research for commercial genetic engineering applications. Reimers sought to capitalize on these advancements in recombinant DNA research by reaching out to several prominent scientists involved in the field, including Berg and Cohen. Berg expressed reservations about patenting academic work. Cohen, initially hesitant, was eventually persuaded by Reimers’ argument that universities should not relinquish the potential financial benefits derived from groundbreaking basic research to private industry. Cohen and Boyer’s strategic decision to pursue a patent application marked a significant milestone for Stanford’s OTL, paving the way for expansion of academic patenting within the field of molecular biology (Hughes 2001; Yi 2011).

On June 24, 1974, Cohen submitted an invention disclosure to Stanford’s OTL, detailing the conceptual origins of recombinant DNA cloning technology.^16^ He attributed the pivotal moment of this invention to a conceptual discussion with Boyer during the Plasmid Meeting in Hawaii in November 1972. According to this invention disclosure, Boyer and Cohen conducted experimental work from the winter of 1972 through the spring of 1973. Cohen cited three important papers published in the *PNAS* in November 1973, April 1974, and May 1974, respectively, as key milestones that demonstrated the feasibility of recombinant DNA cloning technology. The deadline for filing the application was about to expire. Reimers thus prompted Stanford to expedite a patent application, emphasizing the tremendous significance of the invention. He noted that the broader scientific community did not fully grasp the commercial implications of this invention until May 1974, when Morrow’s collaboration with Boyer and Cohen on frog gene cloning was publicly announced (Hughes 2001).

Reimers was well aware of the potential conflicts of interests for Cohen and Boyer, as they already signed the July 1974 Berg letter that called for a halt to recombinant DNA experiments involving animal viruses and certain drug-resistance genes. Reimers underscored Stanford’s awareness of the biohazard risks associated with recombinant DNA technology. In his letter to the NIH’s patent manager, Reimers stated that Stanford “intend[ed] to exercise utmost care in the administration of this invention, within the constraints of the potential patent grant, to prevent its misuse.”^17^ At the pivotal juncture of Asilomar II, the potential of intellectual property rights, specifically patenting, as a regulatory tool for biosafety emerged as a compelling concept, seemingly lending credence to Stanford University’s pending patent application (Creager 1999; Wellerstein 2008).^18^ Reimers, however, did not go so far as to be involved with biohazard discussions, conceding that “the primary safeguard against misuse lies not in patent rights, but rather in the responsible conduct of the international scientific community and their restraint from uncontrolled experimentation.”^19^

Meanwhile, Berg was busy preparing for Asilomar II. He organized a NAS Study Committee, which initially consisted of David Baltimore, James Watson, Dan Nathans, Sherman Weissman, Norton Zinder, and Richard Robin. In the so-called Berg letter, published on July 26, 1974 in *Science*, as well as in *Nature* and *PNAS*, they discussed and called for a voluntary moratorium on two types of recombinant DNA experiments: the first type was the introduction of resistance genes into bacterial species that do not already express that type of resistance; the second type was on the linkage of animal virus genes to plasmids. An experimental plan to link animal DNAs to plasmid DNAs should “not be undertaken lightly” as “many types of animal cell DNA’s contain sequences common to RNA tumor viruses” (Berg et al., 1974, p. 303). The Berg letter in turn called for a meeting of scientists to discuss the potential biohazard risks associated with recombinant DNA technology, which was to be Asilomar II. While they were not members of the Asilomar planning committee, Boyer and Cohen too decided to sign the letter together.

The Asilomar committee, composed of three working groups—the Plasmid Working Group, the Animal Virus Working Group, and the Eukaryotic DNA Working Group—conducted a preliminary assessment of key biohazard concerns associated with recombinant DNA research. Their mandate included identifying potential biohazards, gathering empirical evidence regarding the risks of recombinant DNA research involving bacterial and animal DNA, and developing a classification system for experiments based on their potential biohazard risk (Wright 1994, pp. 145–147). Recombinant DNA experiments involving eukaryotic DNA were deemed the most hazardous, as scientists believed that eukaryotic genomes harbored latent forms of pathogenic viruses, in particular oncogenic viruses (Morange 2020, pp. 197–199). Falkow, who was at the Plasmid Working Group, recalled that the discussions were heavily influenced by the interests of virologists. At the time, as Cohen observed, plasmid biology was considered a marginal field, and its potential significance in the commercial development of recombinant DNA technology was not fully anticipated (Cohen 2009, p. 87). Falkow later complained that “the most stringent prohibitions were being put on the people who worked with microorganisms” (Krimsky 1982, p. 129). Certain experiments with plasmids, due to medical concerns related to antibiotic resistance genes, eventually were placed in the class of experiments prohibited under any circumstances at that time.

The Asilomar planning committee briefly considered inviting some of the scientists who could comment on the ethical and social implications of recombinant DNA technology. One of them was MIT’s Signer, who had been active in discussions about the potential social and moral implications of genetic engineering. Donald Brown, who oversaw the Asilomar’s Eukaryotic DNA Working Group, asked him about the potential risks involving eukaryote-prokaryote hybrid organisms. Signer responded by proposing to discuss the prospects of genetic engineering and its societal implications, including eugenics and control over research direction. Signer was a member of the Genetics and Society Group (GSG), established in December 1974 as a branch of Science for the People, which advocated for a more equitable distribution of the benefits and burdens associated with genetic engineering. The group asserted that decisions regarding who would benefit from and who would bear the risks of this emerging technology should not be solely in the hands of scientists. Signer ultimately was not invited to Asilomar (Krimsky 1982, pp. 99–112; Botelho 2019).

Due to the scientists’ efforts to confine the scope of discussion on the implications of recombinant DNA technology to technical risks, Asilomar II largely focused on technical considerations, particularly those related to the unintentional transfer and cloning of infectious and cancerous genes. As highlighted by Krimsky, at the opening session Baltimore emphasized that two critical areas should be excluded from the deliberations. The first was the application of recombinant DNA techniques for genetic engineering of humans, as these involve intricate ethical values and political considerations that could potentially obscure the technical discussions on biohazards. The second was the potential military utilization of gene-splicing techniques (Krimsky 1982; Cobb 2022).

A few months after the Berg letter called for a research moratorium on recombinant DNA technology, in November 1974 the Stanford OTL proceeded with the patent application for recombinant DNA technology on behalf of Cohen and Boyer. Disputes about patenting and commercial considerations began to emerge among the scientists initially involved in recombinant DNA’s scientific development, complicating their attempt to control biohazard risks. Stanford OTL’s Attorney, Bertram Rowland, in preparation for Stanford’s patent application for gene cloning, sought waivers of co-inventorship from individual co-authors who had collaborated with Cohen and Boyer on their three *PNAS* publications. Morrow, the first author of the cloning of a eukaryotic gene, and Robert Helling, the corresponding author of the first replication of an intra-species gene, contested Stanford’s OTL’s determination of the legal inventors of recombinant DNA technology. Helling and Morrow did not sign these waivers. Both were also taken aback by the patent claims, which excluded them as co-inventors.

In response, Morrow and Helling wrote letters inquiring about Stanford’s patenting endeavors, sparking concern among other Stanford scientists and administrators. On January 23, 1975, Morrow wrote to Berg, who was preparing for Asilomar II. Morrow also wrote to Cohen and Boyer, insisting that he would not sign a patent disclaimer. He was baffled as to why he himself was “not a co-inventor of recombinant DNA plasmids containing DNA foreign to *E. coli* nor of methods for making them.” He further asked how it could be “possible for you to patent these methods and plasmids when the work was funded mainly by the NIH?” He requested a copy of the patent application, raising questions about its broad scope. He eventually insisted that it would be “essential that other scientists carry out research on recombinant DNA molecules without restraint by your patent.”^20^

Helling, in a letter to Rowland, also declined to sign a disclaimer of co-inventorship. But he emphasized that “even the narrowest definition of ‘inventors of the procedures involved' must include, in addition to Boyer, Cohen, and myself, at least J. Morrow, R. Davis, and J. Mertz.” Furthermore, he noted that “similarly the development of a procedure for transforming *E. coli* by Mandel and Higa led them along a similar line of reasoning.” Helling also highlighted that “Boyer was in Europe when the last experiments were performed, and the writing of this paper was completed.” He concluded by stating his intention to “discuss [the matter] further with H. Boyer, S. Cohen, and the others in California in a week.”^21^

Cohen took their letters seriously and wrote to Rowland right before Asilomar. He claimed that the patenting was “pursued at the initiative of the university and that [he] would not receive personal gain from the patent.” His rationale was that “any financial benefits derived from this kind of scientific research carried out at a non-profit university with public funds to go to the university, rather than be treated as a wind-fall profit to be enjoyed by profit-motivated businesses.” Cohen continued to defend this position, saying that he “agreed to cooperate with Stanford for that reason” (Cohen 2013).^22^

In response, on February 10, 1975, Reimers wrote to Stanford administrator William Massy, detailing discussions with Stanford biochemists, especially those who played a major role in the declaration of the voluntary moratorium. He indicated that the “subject was the perception of Stanford’s motives by the scientific community in filing a patent application for the ‘gene transplant’ work, while at the same time advising the scientific community to ‘go slow’ with respect to related research.”^23^ To alleviate concerns, Stanford’s legal counsel stated that neither Cohen nor Boyer would personally benefit financially from the invention. The discussion in Berg’s office explored potential solutions, such as transferring the invention to the Research Corporation, abandoning the patent entirely, or proceeding as planned. Even so, as Matthew Cobb has argued, Berg was concerned that publicizing patenting efforts might derail discussions on the crucial biosafety issues surrounding recombinant DNA technology (Cobb 2022, p. 78). Widespread awareness of the patenting efforts within the recombinant DNA research community did not emerge before and during Asilomar II.

On February 24, Asilomar II began with opening remarks by Baltimore. The three and a half-day conference focused primarily on presentations of experiments conducted with recombinant DNA technology, with one session devoted to accessing its legal and social implications. The participants at Asilomar II still sought to establish a consensus regarding the risks associated with these novel experimental techniques and the corresponding precautionary measures to be implemented. During the meeting Helling “asked Cohen about the patent application. He refused to discuss the matter with me in any way,” Helling recalled.^24^ Cohen’s patenting application seemed to be known there. A *Rolling Stone* article depicted a man “like a newly busted big-time mobster hiding behind his fedora on the steps of a precinct house,” and Cohen later admitted that he was the man referred to in the article (Rogers 1975; Cohen 2009, p. 90). After Asilomar II, some scientists raised ethical concerns about the potential for individual institutions or scientists to profit from taxpayer-funded research. They questioned whether hasty patent filings aligned with “the public service ideals of the University.”^25^

In a significant development, the US Patent and Trademark Office granted most recombinant DNA process claims in its initial decision on March 31, 1975. This decision, while not granting patents for recombinant products, served as a catalyst for the commercialization of genetic engineering. Boyer, with his entrepreneurial spirit, recognized the immense potential of recombinant DNA technology. As early as 1974, he had considered the production of hormone pharmaceuticals such as human insulin and angiotensin-2 through gene cloning (Rasmussen 2014). The favorable patent ruling provided the impetus for Boyer to found Genentech with young venture capitalist Robert Swanson in April 1976. Cohen, in the meantime, was heavily involved in opposing legislative control of recombinant DNA research, and exhibited a notable reluctance to engage directly in commercial ventures that would compromise his credibility. Rather than join Genentech, Cohen served only as a scientific advisor to Cetus, a biotech firm recently established in Berkeley (Cohen 2009, p. 155).

Cohen’s involvement in patenting efforts amid ongoing biohazard concerns placed him in a defensive position, compelling him to claim that Stanford’s patent rights could serve as a means of mitigating potential biohazards associated with recombinant DNA technology (presumably by withholding or withdrawing licenses for those who would undertake hazardous research). At the Miles symposium held at MIT between June 8th and June10th, 1976, someone asked if DNA cloning was being patented. Some scientists even expressed concern over whether Cohen’s private ownership of recombinant DNA technology hindered its use in a wide array of biomedical research; others accused him of attempting to control DNA-cloning research through the patent filing.^26^ Baltimore, who was chairing the session, asked Cohen if he would like to comment on that matter. Cohen said that Stanford intended to require industrial licensees to adhere to the NIH guidelines. He continued that this patent allowed the university to ensure the biosafety of recombinant DNA research (Cohen 2009, p. 152). Faced with public criticisms from his fellow researchers, Cohen conceded to Reimers that “while I am not proposing right now that we call the whole [patenting] thing off, I would have no objection to such a decision by the University”^27^ (Yi 2015).

## Regulation and the Commercial Fate of Gene Cloning

In June 1976, the NIH convened a public hearing to gather input on a proposed set of guidelines. One of the key concerns raised during the hearing was the potential for conflicts of interest among scientists involved in developing the guidelines. Roy Curtiss, a prominent scientist in the field, pointed out that researchers might be regarded as having vested interests in the specific recommendations due to their involvement in recombinant DNA experiments. To address these concerns, he suggested that the NIH seek input from an inclusive group of individuals representing not only scientific disciplines but also other civic society groups. The public hearing revealed a range of dissenting opinions regarding the proposed NIH guidelines. Historian Susan Wright categorized these dissenters into three main groups: those concerned about the proliferation of recombinant DNA experiments without sufficient knowledge of potential risks; those concerned about non-government-sponsored research that would not be required to abide by the NIH guidelines; and those calling for greater public representation in regulatory discussions (Wright 1994, p. 183). Despite hearing these concerns, the NIH published recombinant DNA guidelines along the lines recommended by scientific experts in July 1976.

The public soon raised a broad set of concerns about genetic engineering work and its commercialization. In March 1977, the National Academy of Sciences organized an Academy Forum to discuss one of the key national policy issues in science and society, namely the regulation of recombinant DNA technology. The forum ignited a heated debate that extended to lay activists. Led by prominent figures such as Jeremy Rifkin, they disrupted the three day event in Washington, DC, expressing concerns about the potential ethical and societal implications of genetic engineering. Their protests, characterized by chants such as “we will not be cloned,” reflected the broader anxieties about the emerging field. The Coalition for Responsible Genetics, a group backed by environmentalists and renowned scientists like George Wald and Sir MacFarlane Burnett, emerged as another key player in the debate (Wright 1994, pp. 221–225). The coalition expressed concerns about the potential biohazards associated with genetic engineering and argued that commercial interests might undermine the effectiveness of the NIH guidelines, as there would be many privately-funded initiatives in genetic engineering that were out of its scope. They protested with a sign that read “public debate before private profit” (Hopson 1977, p. 57).

While Stanford suggested that the control of private recombinant DNA activities through patent licensing would ensure its safe use according to the NIH regulation, in 1977 a rumor circulated about a laboratory in California that had allegedly violated the NIH guidelines on gene cloning. A reporter called Boyer to confirm that UCSF scientists created “bacterial virus which couldn’t be killed […] had been created by accident” (Hopson 1977, p. 62). The incident came to light when the UCSF administration investigated in response to the rumors. In fact, Axel Ullrich, a Postdoctoral Fellow in the Laboratory of Howard Goodman at UCSF mistakenly considered that the pBR322 plasmid was “approved” for use, not knowing that it had yet to be “certified” for use in an experiment. Primarily working on the rat insulin gene cloning project, Ullrich had obtained pBR322 from Boyer’s Laboratory and performed the initial cloning experiments with pBR322 and rat insulin cDNA from January 16 to March 3, 1977. Clones with recombinant rat DNA had been successfully generated, and even after UCSF scientists found out the breach of the NIH guidelines on February 4, they continued to work with the clones, demonstrating that one contained insulin-specific sequences. The successful rat cDNA clones were eventually destroyed.^28^ Later, there emerged the suspicion that the insulin gene experiment published in *Science* might not have been performed as described, as the experiment had been completed only three weeks after the NIH’s certification of the pMB9 plasmid vector that the paper described using (Rasmussen 2014, pp. 55, 62). In an article in *Science*, UCSF microbiologist David Martin even insisted that “capitalism sticking its nose into the lab has tainted interpersonal relations—there are a number of people who feel rather strongly that there should be no commercialization of human insulin” (Wade 1977, p. 1342). Indeed, an ethnographic participant–observer study in Boyer’s Lab during this period reported that there was an intensely competitive spirit among the young scientists racing to clone the insulin gene, and that for them “it seemed almost chic not to know the NIH rules” (Hopson 1977, p. 62).

The concerns about biosafety and the construction of a regulatory regime to manage it in the mid- and late-1970s influenced Genentech’s business strategies. By mid-1976, Genentech received a $100,000 venture capital investment from Kleiner-Perkins, which was used for work on the insulin gene cloning project—in Boyer’s laboratory. Goodman, along with another competitor, Walter Gilbert at Harvard University, also tried to clone human insulin DNA, experiments requiring a P4-Level Laboratory. Boyer, after witnessing regulatory breaches derailing experimental projects and reluctant to work in a P4 Facility, decided to seek another avenue in his pursuit of the insulin gene cloning. Along with the founding of Genentech, that decision strained the “family-like collaboration” Boyer had enjoyed with Goodman (Hall 1987, p. 92). On behalf of Genentech, Boyer sought to avoid the regulatory hurdles associated with natural DNA cloning by adopting a novel strategy involving the synthesis of artificial genes. This approach bypassed the NIH guidelines (which were driven by the risks associated with introducing unknown animal and virus gene fragments into live bacteria) and opened up new possibilities for recombinant DNA. Thus Boyer contacted synthetic DNA experts Arthur Riggs and Keiichi Itakura, of the City of Hope Hospital in Southern California, to arrange a contract project to synthesize an artificial insulin gene in February 1977 (Hughes 2011; Rasmussen 2014). As Riggs later recollected, the recombinant DNA controversy “actually influenced a change in the plan.”^29^

In December 1977, Genentech’s scientists announced the cloning of a human hormone, somatostatin, demonstrating their synthetic gene approach could be used for making medically useful proteins (Itakura et al. 1977). The resulting Riggs–Itakura patents involving the use of artificial genes for recombinant DNA technology were indeed fundamental to the biotech industry. In September 1978, scientists at Genentech, in collaboration with Riggs and Itakura, inserted their artificial insulin gene into a plasmid and cloned it in order to demonstrate the production of pure bacterially-produced insulin protein. Prompted by this success, Eli Lilly signed a contract with Genentech. By late 1978, the NIH began relaxing its guidelines for recombinant DNA work on higher organisms, considering recent developments in science, such as the discovery of introns, which suggested that bacteria did not have the complex machinery to express natural eukaryotic genes. Genentech’s press conference, which was held on September 6, 1979, announced the production of medicine using genetic engineering without the biohazards underlying the NIH guidelines, signaling that the age of biotechnology had finally arrived (Hughes 2011; Rasmussen 2014).

## Conclusion

The advent of recombinant DNA technology revolutionized molecular biology, progressively placing gene cloning at the forefront of biomedical research and fundamentally altering the field’s central activity from representation to a deliberate rewriting of life (Rheinberger 1995). By early 1980s, molecular biologists and nascent biotechnologists raced to isolate, amplify, and clone eukaryotic genes, making *Molecular Cloning: A Laboratory Manual* a popular reference (Creager 2020). At the beginning, Berg’s Group at Stanford occupied a leading position in recombinant DNA research, with graduate students like Lobban, Mertz, and Morrow contributing significant advancements in the biochemical manipulation of genetic material. Their contributions broadened the scope of recombinant DNA technology, both in gene cloning and its application to genetic engineering, thereby establishing its technological feasibility. While Signer and Mertz initially raised the societal concerns regarding genetic engineering, particularly the delicate balance between its benefits and risks, these concerns were largely confined to discussions of public health risks associated with cancer viruses. The voluntary moratorium implemented by the Berg Group in 1971, however, was subsequently eclipsed by rapid and unforeseen breakthroughs in recombinant DNA research. Mertz’s discovery of cohesive ends of *Eco*RI significantly simplified recombinant DNA procedures, while a series of gene cloning experiments conducted by Cohen, Boyer, and Morrow garnered substantial attention due to the perceived commercial potential of this technology.

Asilomar II constituted a new attempt to manage both the enthusiasm and apprehension surrounding recombinant DNA technology, with discussions centered on whether to lift the existing voluntary moratorium and how to balance the potential benefits and risks of gene cloning. Morrow’s cloning of animal genes, coupled with Stanford’s patenting of recombinant DNA technique, marked a turning point, shifting the primary focus of leading molecular biologists from public health concerns towards proprietary and commercial interests. News of Stanford’s patent application circulated even at Asilomar, and the decision to request NIH oversight of recombinant DNA technology significantly empowered scientists to control the future of gene cloning. From the mid-1970s onward, two sets of initiatives promoting deregulation and private ownership within genetic engineering gained political momentum. These initiatives, designed to enhance the individual rewards associated with the commercialization of gene cloning, ultimately contributed to the relaxation of NIH guidelines in recombinant DNA technology and the enactment of the Bayh–Dole Act of 1980, which encouraged universities to patent federally-funded research (Wright 1994; Yi 2011).

Many leading American scientists, who faced restrictions on recombinant DNA experiments involving DNA from animal viruses and higher organisms, embarked on a competitive race to clone the insulin gene after Asilomar II. Success in this contest, which involved Gilbert’s Biogen, Goodman’s UCSF laboratory, and Boyer’s Genentech, hinged on their ability to navigate and, in some cases, circumvent existing regulations governing eukaryotic gene cloning. Biogen, embroiled in a biosafety controversy in Cambridge in 1976, was limited to cloning rat genes within a P4 Containment Laboratory in Britain. While Goodman’s Laboratory successfully cloned the rat insulin gene, allegations of NIH regulatory violations raised questions regarding the patentability of their insulin clones. Ultimately Genentech, employing a synthetic gene approach, was regarded as the winner of the race to clone the human insulin gene (Rasmussen 2014, pp. 59–66). But if not for Asilomar, I have argued here, that outcome might have been different.

By the late 1970s, the discourse surrounding responsible research practices and biosafety in genetic engineering had been supplanted by critiques concerning the impact of regulations and legal frameworks on scientific competition and laboratory culture. Morrow’s baffling questions regarding intellectual property ownership in gene cloning became increasingly pertinent with its commercialization. Despite their essential scientific contributions, neither Mertz nor Morrow received legal recognition as inventors of recombinant DNA technique. While the moratorium’s underlying intention was laudable, its implementation as a broad measure failed to address the emerging commercial motivations and the evolving legal landscape of biotech entrepreneurship, necessitating sacrifices from early pioneers in the field. Indeed, as historian Nicolas Rasmussen astutely observes, the widespread acclaim and financial success achieved by Genentech through the cloning of the insulin gene highlighted the potential for “commercial interests penetrating science’s own system of intellectual credit,” commercial success and patent recognition overshadowing the credit accorded to other crucial academic contributions, i.e., the elucidation of insulin gene function and the mechanisms of eukaryotic gene expression (Rasmussen 2014, p. 66). The molecular biology leaders who convened at Asilomar subsequently had to grapple with the profound transformative impact of burgeoning proprietary interests on the moral and political economy of their field (Kohler 1994; Yi 2015).

## Data Availability

No datasets were generated or analyzed during the current study.

## Footnotes

1 Janet E. Mertz, Interview, March 9, 1977. Oral History Programs, Recombinant DNA Controversy Collection, MC 100, MIT Institute Archives (hereafter, Mertz interview).

2 *The New York Times,* “Playing with Biological Fire,” December 8, 1969.

3 Mertz i, p.7.

4 Mertz interview, p. 7.

5 Paul Berg to R. Pollack, “To Registrar of Courses,” March 15, 1971, Box 3, Folder “General Correspondence (all 1970 and Jan-June 1971). Paul Berg Papers, University Archives SC 358, Stanford University, Stanford, California (hereafter, Berg Papers).

6 Mertz interview, p. 17. 

7 Mertz interview, pp. 4–5. I appreciate Luis Campos for pointing out this quote to me.

8 Mertz interview, p. 9.

9 Mertz interview, p. 20.

10 Mertz interview, p. 34.

11 S. Falkow Declaration under Rule 132, 22 October 1982 in Finnegan, Henderson, Farabow, Garrett & Dunner, LLP, (1985) Opinion Regarding Validity, Enforceability and Infringement Issues Presented by the Cohen and Boyer Patents. Appendix, Exhibits #15; SUOTL

12 Paul Berg, “A Stanford professor’s career in biochemistry, science politics, and the biotechnology industry.” An oral history conducted in 1997 by Sally Smith Hughes. Regional Oral History Office, Bancroft Library, University of California, Berkeley, Berkeley, California (hereafter, Berg oral history), pp. 105–106; and see Stephanie Chen, 2016. “2013 06 April John Morrow Interview.” Retrieved from https://hdl.handle.net/10161/11702, December 29, 2024.

13 Janet. Mertz to M. L. Pardue, November 17, 1973, Janet E. Mertz Papers, Box 4, CSHL Archives, Cold Spring Harbor Laboratory, Cold Spring Harbor, New York.

14 Berg oral history, p. 106.

15 Berg oral history, p. 120.

16 Stanley N. Cohen, Stanford University Invention Disclosure, “A Process for Construction of Biologically Functional Molecular Chimeras, Inventor/s S.N. Cohen, H.W. Boyer,” SEL Form No. 207, signed and dated by Stanley N. CohenJune 24, 1974 Box 95, Folder 3, “Cohen/Boyer Patent Information,” Series 11, Stanley N. Cohen Papers, Manuscript Collection 623, National Library of Medicine, Bethesda, Maryland (hereafter, Cohen Papers)/

17 N. J. Reimers to N. J. Latker, August 20, 1974, Box 95, Folder 3, Series 11, Cohen Papers.

18 Patenting has been justified as a means of controlling product quality in pharmaceuticals and safeguarding national security in nuclear technologies.

19 Reimers to Latker, August 20, 1974, Box 95, Folder 3. i Series 11, Cohen Papers.

20 J. Morrow to S. Cohen and H. Boyer, January 23, 1975 i Stanford University Office of Technology Licensing Archive, Stanford, California (hereafter, SUOTL), S74, 43.

21 R. Helling to B. Rowland, February 11, 1975 in Finnegan, Henderson, Farabow, Garrett & Dunner, LLP(1985) *Opinion Regarding Validity, Enforceability and Infringement Issues Presented by the Cohen and Boyer Patents*. Appendix, Exhibits #16; SUOTL.

22 Stanley. N. Cohen to B. Rowland, January 22 1975, Folder, “Correspondence 1974-1979.” SUOTL, S74-43.

23 Reimers to W. Massy, February 10, 1975, S Box 95,Folder 4, Series 11, Cohen Papers

24 R. Helling to B. Rowland, March 16, 1983 in Finnegan, Henderson, Farabow, Garrett & Dunner, LLP, 1985. *Opinion Regarding Validity, Enforceability and Infringement Issues Presented by the Cohen and Boyer Patents*. Appendix, Exhibit Item #16; SUOTL.

25 “In Confidence, Re: Process and Composition for Biological Functional DNA Chimeras,” July 1, 1975, Correspondence 1974–1979, SUOTL.

26 See “Genetic Manipulation to be Patented?” *Nature* 261 (1976): 624.

27 Cohen to Reimers, June 14, 1976, SUOTL.

28 Report to the Office of Recombinant DNA Activities concerning Premature Use of pBR322 at UCSF during 1977, January 20, 1978, Box 2, Folder 24, Series 2, William J. Rutters Papers, Manuscript Collection MSS 94-54, University of California, San Francisco, San Francisco, California.

29 Arthur D. Riggs, “City of Hope’s contribution to early Genentech research.” An oral history conducted in 2005 by Sally Smith Hughes. Regional Oral History Office, The Bancroft Library, University of California, Berkeley, Berkeley, California, p. 46.
